# P-239. Leveraging Preliminary Blood Culture Reports at an Academic Medical Center: A Real-time Central Line-Associated Bloodstream Infection (CLABSI) Reduction Strategy

**DOI:** 10.1093/ofid/ofae631.443

**Published:** 2025-01-29

**Authors:** Meghan Hudziec, Kathryn Rice, Barbara Wenger, Sarah Elizabeth Totten, Larissa Pisney

**Affiliations:** University of Colorado Hospital, Denver, Colorado; University of Colorado Hospital, Denver, Colorado; University of Colorado Hospital, Denver, Colorado; University of Colorado Hospital, Denver, Colorado; University of Colorado School of Medicine, Aurora, Colorado

## Abstract

**Background:**

CLABSIs have been shown to result in adverse outcomes including increased morbidity and mortality, length of hospital stays, and healthcare costs. The National Healthcare Safety Network (NHSN) surveillance criteria are meant to be applied in a standardized manner, but this can result in discordance between clinical diagnoses and the surveillance definitions of secondary bloodstream infections (2° BSI). We hypothesized that routine review of preliminary positive blood cultures (B/Cs) in patients with central lines would result in the identification of possible CLABSI events in real-time and promote dissemination of feedback, thus reducing CLABSI cases and increasing 2° BSI attribution.

**Figure 1:**
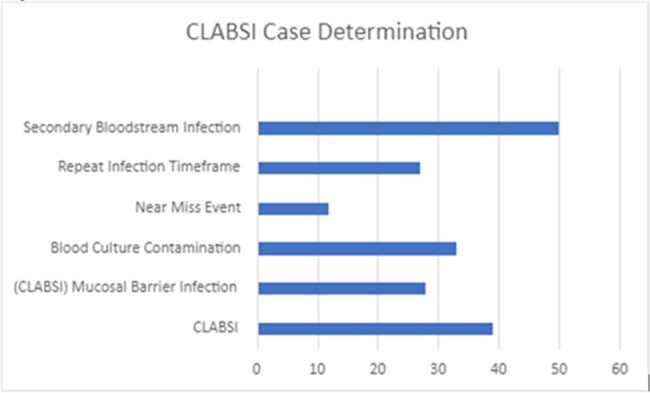
CLABSI Case Determination Classification of cases identified by review of a preliminary blood culture report for patients with a central line eligible for CLABSI surveillance

**Methods:**

An automated electronic medical record (EMR) report was developed to capture preliminary B/C results for patients with a central line eligible for CLABSI surveillance. Daily EMR review was performed to identify opportunities contributing to the event or evidence of a clinical 2° BSI without corresponding elements needed to meet NHSN definitions. Possible B/C contamination, gaps in care and, if clinically appropriate, necessary elements required to meet NHSN 2° BSI definitions were communicated to the care team.

Secondary BSI Attribution

**Figure:**
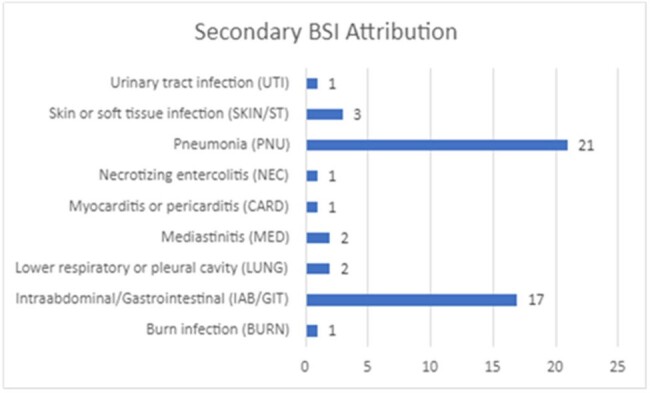

Categorization of cases with a final determination of secondary BSI attribution

**Results:**

Confirmed CLABSI cases with no intervention indicated were 35.5% of cases reviewed; 38.1% were determined to meet CLABSI exclusion criteria. Evidence of 2° BSI was identified in 26.5% of cases, which prompted queries to the care team to consider additional imaging, cultures, or documentation needed to clarify the clinical assessment. Feedback related to possible B/C contamination was given in 17.5% of cases. (Fig. 1-3)

CLABSI Standardized Infection Ratio (SIR)

**Figure:**
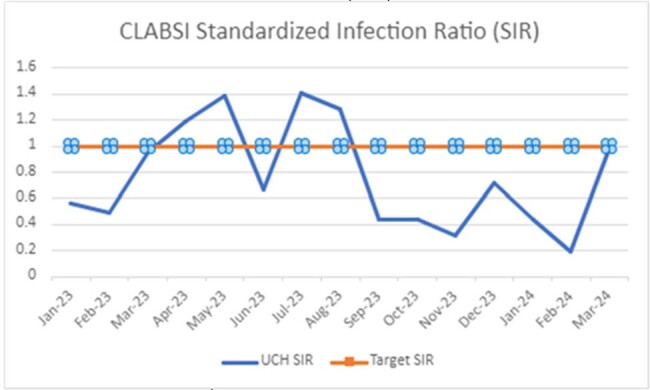

Standardized infection ratio (SIR) trend: Tactic was adopted in May 2023

**Conclusion:**

CLABSI reduction requires a comprehensive approach that addresses various aspects of care including appropriate line selection, safe insertion practices, care bundles, and prompt line removal. The EMR can be used to hard-wire best practices, facilitate real-time communication, and identify CLABSI cases in a timely manner. We have shown that prompt review of positive B/Cs of patients with eligible central lines facilitates opportunities to align the clinical impression with the surveillance definitions for 2° BSI while bolstering quality improvement efforts and staff education.

**Disclosures:**

**All Authors**: No reported disclosures

